# Randomized Oblivious Transfer for Secure Multiparty Computation in the Quantum Setting

**DOI:** 10.3390/e23081001

**Published:** 2021-07-31

**Authors:** Bruno Costa, Pedro Branco, Manuel Goulão, Mariano Lemus, Paulo Mateus

**Affiliations:** 1 Departamento de Matemática, Instituto Superior Técnico, Av. Rovisco Pais, 1049-001 Lisbon, Portugal; brunofilipe.antunescosta@capgemini.com (B.C.); pedrodemelobranco@gmail.com (P.B.); manuel.goulao@tecnico.ulisboa.pt (M.G.); marianojlemush@gmail.com (M.L.); 2 Capgemini Engineering, Av. D. João II, Lote 1.07.2.1, Piso 2, 1990-096 Lisbon, Portugal; 3 Instituto de Telecomunicações, IST Av. Rovisco Pais, 1049-001 Lisbon, Portugal

**Keywords:** oblivious transfer, quantum cryptography, post-quantum cryptography, universal composability

## Abstract

Secure computation is a powerful cryptographic tool that encompasses the evaluation of any multivariate function with arbitrary inputs from mutually distrusting parties. The oblivious transfer primitive serves is a basic building block for the general task of secure multi-party computation. Therefore, analyzing the security in the universal composability framework becomes mandatory when dealing with multi-party computation protocols composed of oblivious transfer subroutines. Furthermore, since the required number of oblivious transfer instances scales with the size of the circuits, oblivious transfer remains as a bottleneck for large-scale multi-party computation implementations. Techniques that allow one to extend a small number of oblivious transfers into a larger one in an efficient way make use of the oblivious transfer variant called randomized oblivious transfer. In this work, we present randomized versions of two known oblivious transfer protocols, one quantum and another post-quantum with ring learning with an error assumption. We then prove their security in the quantum universal composability framework, in a common reference string model.

## 1. Introduction

Oblivious transfer (OT), first introduced by Rabin in 1981 [1], is an important primitive in modern cryptography. The OT primitive is known to be a basic building block for other cryptographic tasks, including secure Multi-Party Computation (MPC), Bit Commitment (BC), Coin-Tossing, and Zero-Knowledge Proofs [2,3,4,5,6,7].

A 1-out-of-2 OT protocol [8] consists of two parties, a sender with two input messages $\left(m_{0},m_{1}\right)$ and a receiver with a choice bit b∈{0,1}. The goal of the protocol is to output only the message $m_{b}$ to the receiver, with no information about $m_{1-b}$, and the sender remains oblivious to the receiver’s input bit *b*. Note that, in the original work by Rabin, called all-or-nothing OT [1], the sender has a single input message, while the receiver has none. The protocol outputs the message to the receiver with probability $\frac{1}{2}$, such that the receiver has no information whether or not the receiver obtained the message. It was shown that one can construct 1-out-of-2 OT from all-or-nothing OT [9]. Another OT variant is that of Randomized Oblivious Transfer (ROT), where neither of the parties have any inputs. The ROT protocol, instead, outputs the messages $\left(m_{0},m_{1}\right)$ to the sender and $\left(b,m_{b}\right)$ to the receiver, with $\left(m_{0},m_{1},b\right)$ chosen uniformly at random from their domains.

MPC [10,11], which is an extremely useful cryptographic tool to compute arbitrary functionalities, can be reduced to the OT primitive; i.e., having access to a secure OT is sufficient [2]. MPC implementations based on oblivious-circuit evaluation techniques require a large number of OT (one per input wire for Yao [10], and one per AND gate for GMW [11]). Since classical OT schemes (being based on asymmetric-key cryptography) are relatively slow, the development of large-scale MPC implementations has been severely hindered by the required OT rates. In order to deal with this issue of OT efficiency, the concept of OT extension was introduced by Ishai et al. in 2003 [12]. This technique refers to extending a small number of computationally expensive base OTs into a larger number of OTs, using only cheap symmetric cryptography primitives. For proving the security of these OT extension techniques in the malicious-adversary setting [13], it turns out that one is required to use ROT instances as the base OTs. Additionally, ROT finds direct application in designing efficient Private Set Intersection (PSI) protocols [14], one of the most popular MPC techniques.

Moreover, even though the efficiency issue can be solved by the use of OT extensions for MPC applications, there is the underlying threat that asymmetric-key based schemes (e.g., integer-factorization or discrete-logarithm problems) will be faced with the arrival of quantum computers [15]. The research initiatives for developing quantum-resistant solutions have been following two paths. The first being on the development of more hard-to-break classical cryptography algorithms that will remain secure even against a quantum adversary. These solutions include the approximate Shortest Vector Problem (SVP) on ideal lattices [16], the Learning with Errors (LWE) problem [17] and its ring version, Ring Learning with Errors (RLWE) [16], constituting a new area of research, called post-quantum cryptography. The second approach is that of quantum cryptography, where solutions for Quantum Key Distribution (QKD), BC, and OT already exist [18]. While unconditional security for QKD has been proven [19], there are impossibility results to achieve for the case of BC and OT [20,21,22]. Nevertheless, practical solutions for BC and OT were proposed under the assumption of physical limitations on the devices, such as noisy storage and bounded quantum memories [23,24,25,26,27].

### Our Contribution

In this work, we explore the construction of two ROT protocols in the quantum Universal Composability (UC) framework, in the Common Reference String (CRS) model:

- A quantum protocol based on the UC construction by Unruh [28] and augmented with an additional subroutine to enforce randomized outputs.
- A classical protocol based on a variant of the RLWE assumption that adapts the one presented in [29,30] but does not require a random oracle model and, instead, uses a composable commitment scheme and a composable non-interactive zero knowledge (NIZK) protocol.

In both cases, the basic idea is to build upon existing non-randomized OT protocols in such a way as to force the values of all of the protocol’s outputs to be influenced by both parties. This allows us to randomize both the messages $m_{0},m_{1}$ and the choice bit *b* as long as at least one party is honest, leading to a ROT protocol. Furthermore, we prove that the resulting protocols are secure in the quantum UC framework.

This paper is organized in five sections. In Section 2, we briefly review some definitions and functionalities relevant for the description and analysis of the protocols. In Section 3, we present the generic construction of ROT from OT and afterwards present the commitment scheme and OT protocols that we will be using to achieve the quantum security we need. The security of the protocols are then shown in Section 4. Finally, in Section 5, we present the main results of this work.

## 2. Background

The problems regarding Ring Learning with Errors are conjectured to be hard on both classical and quantum computers. Before defining the RLWE distribution and its decision problem, we first present the notation used. Let $R_{q}=Z_{q}\left[X\right]/f\left(X\right)$ be a ring, where q>2 is a prime, and f(X) is a cyclotomic polynomial of degree *n*. Let β∈N and χ be the error distribution that outputs elements of $R_{q}$ with a norm greater than β with negligible probability.

**Definition** **1** (RLWE distribution)**.** *Let $q,R_{q}$ and χ be as above. The RLWE distribution $A_{s,\chi }$ is obtained by sampling $a\in R_{q}$ uniformly, choosing e←$ χ and outputting (a,b=as+e mod q) for a secret $s\in R_{q}$.*

**Definition** **2** (decision-RLWE)**.** *Let $q,R_{q},\chi$ and $A_{s,\chi }$ be as above. For s←$ R_q_, given many polynomial samples, the goal is to distinguish between $A_{s,\chi }$ and a uniform distribution over R_q_ × R_q_.*

By using the the RLWE variant of the LWE problem we are able to not only work with smaller keys but also increase the speed of the operations by using the Number Theoretic Transform (NTT). The protocol we will be analyzing uses a variant of the RLWE problem, the Hermite Normal Form of the RLWE problem (HNF-RLWE), in which the secret *s* is sampled from the error distribution *χ* instead of being chosen uniformly at random from the ring $R_{q}$. This version of the problem is assumed to be hard as well, since RLWE reduces to it [31].

Often times studying the standalone security of protocols is not enough, since they will be frequently used as subroutines in more complex tasks, as is the case of OT, as well as Coin Tossing, Commitment schemes, Zero-Knowledge proofs, etc. In order to ensure that protocols are secure in any computational environment, Canetti [32] introduced the Universal Composability (UC) framework, which we define next.

Let *π* be an n-party protocol and F be an ideal functionality. We denote as ${\mathrm{IDEAL}}_{F,S,Z}$ the output of the environment Z at the end of the ideal-world execution of functionality F with adversary S, and as ${\mathrm{EXEC}}_{\pi ,A,Z}$ the output of the environment Z at the end of the real-world execution of *π* with adversary A. The notion of a protocol securely emulating some ideal functionality is as follows:

**Definition** **3** (UC-secure)**.** *We say that π UC-emulates F if for any adversary A there exists a simulator S, such that, for all environment Z,*

$$\begin{array}{c} {\mathrm{IDEAL}}_{F,S,Z}\approx {\mathrm{EXEC}}_{\pi ,A,Z}. \end{array}$$

When discussing UC security, we can consider either a bounded (computational) or unbounded (statistical) approach. In computational UC security, we restrict the adversary, simulator, and environment to polynomial-time machines, and this approach is used when showing security based on computational assumptions. On the other hand, in statistical UC security, we quantify over all adversaries, simulators, and environments; as such, we can model statistical security.

In this work, we consider malicious adversaries, that is, adversaries that can deviate in any way from the protocol. However, we assume that the corruption of a party happens before the start of the protocol, and both the sender or the receiver may be corrupted.

**Figure 1:**
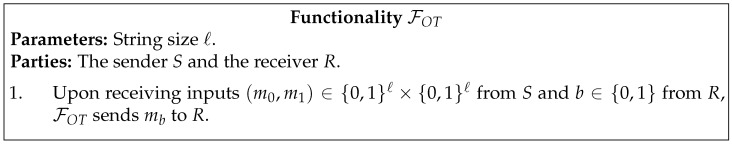
OT functionality.

**Figure 2:**
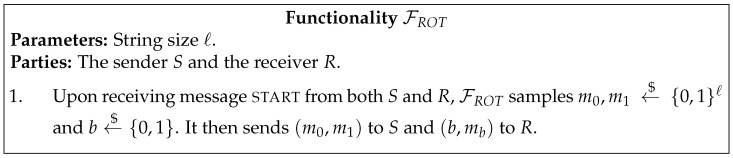
ROT functionality.

**Figure 3:**
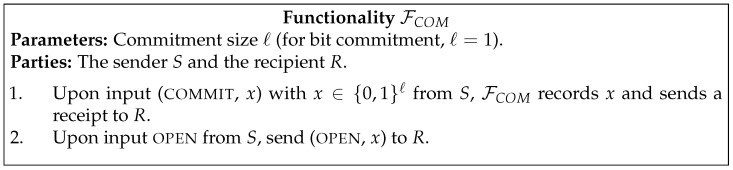
Commitment functionality.

**Figure 4:**
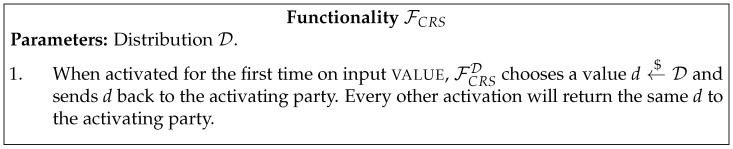
Common Reference String functionality.

**Figure 5:**
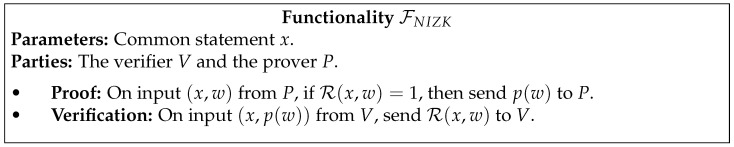
Non-Interactive Zero-Knowledge functionality.

In Figure 1, Figure 2, Figure 3, Figure 4 and Figure 5 we present the functionalities that will be relevant in this work.

We stress that the definition of $F_{ROT}$ presented here is stronger than the one presented in Unruh’s original paper [28], in which the outputs are only random if the parties are both honest. In the same paper, the UC framework is extended to the quantum setting by allowing the protocol *π*, the adversary A, the simulator S, and the environment Z to be quantum.

Unruh [28] also showed that, when *π* is a classical protocol and *π* statistically UC-emulates F, then *π* statistically quantum-UC-emulates F, providing a lift from statistical classical-UC to statistical quantum-UC. A similar result exists for the computational case [28], but it is required that the adversary in the classical case is given the same computational power as in the quantum setting; in other words, we need to guarantee that the classical machines present in the proof of UC security are as powerful as quantum-polynomial-time machines.

Consider protocols *π* and *σ*, we denote the protocol where *σ* invokes instances of *π* by $\sigma^{\pi }$. A usual situation would be $\sigma^{F}$, being a protocol that uses some ideal functionality F, and $\sigma^{\pi }$ would then be the protocol that results from implementing that functionality with some protocol *π*. Composition has been shown to be secure, both in the classical [32] and quantum settings [28].

**Theorem** **1** (Universal Composition Theorem [28])**.** *Let F,G be ideal functionalities. Let π be an n-party protocol that UC-emulates G in the F-hybrid model, and let η be an n-party protocol that UC-emulates F. Protocol $\pi^{\eta }$ then UC-emulates G.*

## 3. Protocols

In this section, we start by presenting the generic construction of ROT from OT, using a commitment scheme, and afterwards describe the commitment scheme and the quantum OT protocol that will allow our ROT protocol to computationally quantum-UC-emulate $F_{ROT}$. Finally, we describe a post-quantum approach, a ROT protocol based on the RLWE assumption, inspired by the recent work of [30], with a small tweak to avoid using random oracles, which misbehave against quantum adversaries.

### 3.1. Generating an UC-Secure Random OT

**Figure 6:**
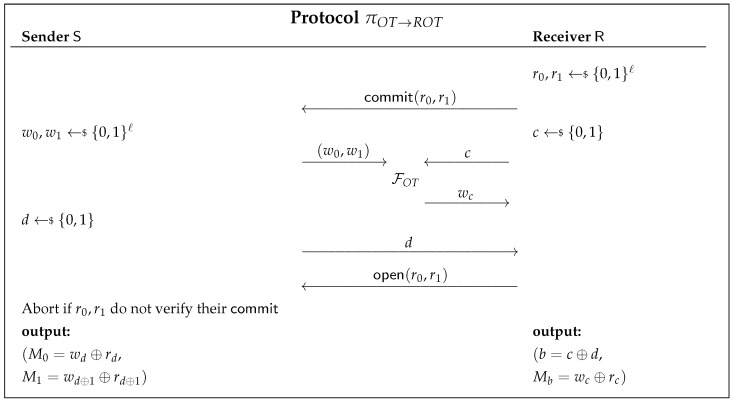
ROT protocol based on secure commitments.

The protocol $\pi_{OT\to ROT}$ is presented in Figure 6. We consider the two parties: the sender S and the receiver R. It begins with R sampling two strings $r_{0},r_{1}\in {\left\{0,1\right\}}^{\ell }$ and committing them to S. R then chooses a random bit *c*, and S chooses two random strings, $w_{0},w_{1}\in {\left\{0,1\right\}}^{\ell }$. With these, the parties invoke the $F_{OT}$ functionality. Following that, S chooses a random bit *d* and sends it over to R. Finally, R opens his commitment, and S checks if it matches the initial commit. If it does not, it aborts; otherwise, it outputs $\left(M_{0}=w_{d}⊕r_{d},\;M_{1}=w_{d⊕1}⊕r_{d⊕1}\right)$. R outputs $\left(b=c⊕d,\;M_{b}=w_{c}⊕r_{c}\right)$.

### 3.2. UC-Secure Commitment Scheme

Canetti [33] showed that UC-secure commitment schemes are impossible in the plain model, and the same result was later proven for the quantum setting as well [22]. With that in mind, we will be working on the Common Reference String (CRS) model defined in Figure 4.

**Figure 7:**
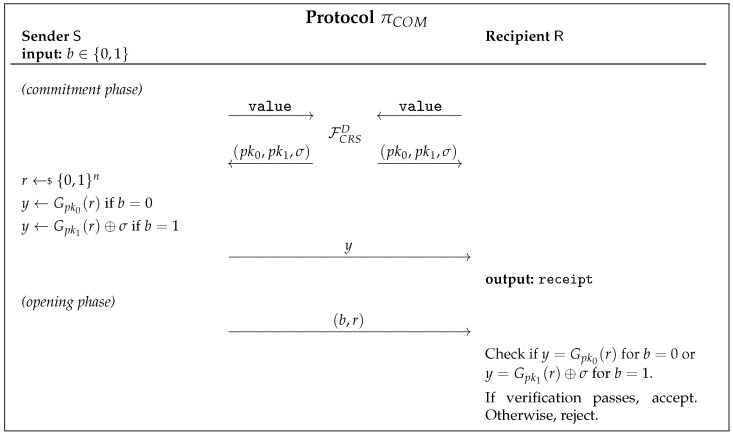
UC-secure BC scheme in the One-Time CRS Model [32].

The protocol $\pi_{COM}$ in Figure 7 has been shown to be computationally UC-secure in the CRS model [33]. The key to this protocol’s composability is the use of a trapdoor pseudo-random generator (PRNG) $G_{pk}$, which is described by its public key pk. This generator $G_{pk}$ stretches *n*-bit inputs to 4*n*-bit outputs, and has a trapdoor td. Having access to both pk and td, we can easily check if a given string $y\in {\left\{0,1\right\}}^{4n}$ is in the range of $G_{pk}$.

Note that the protocol $\pi_{COM}$ is a bit commitment protocol, and for string commitment, an instance of $\pi_{COM}$ is needed to run for each bit of the string.

### 3.3. UC-Secure Quantum OT Protocol

**Figure 8:**
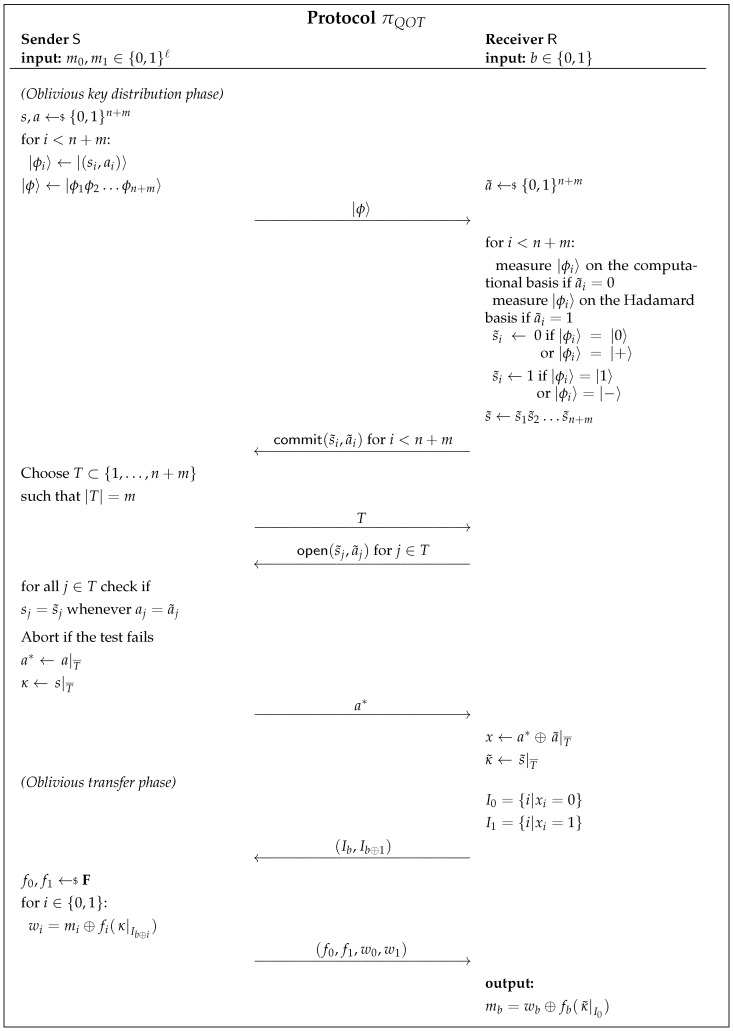
Quantum UC-secure Quantum OT Protocol based on secure commitments [28].

The protocol in Figure 8 was proposed by Yao and has been shown to be statistically quantum-UC-secure with ideal commitments [28].

We describe the logical qubit states |0〉 and |1〉 (representing the computational basis), and the states $|+\rangle =(|0\rangle +|1\rangle )/\sqrt{2}$, $|-\rangle =(|0\rangle -|1\rangle )/\sqrt{2}$ (representing the Hadamard basis). We use the following notation to define the states $|(s_{i},a_{i})\rangle$ for $s_{i},a_{i}\;\in \;\left\{0,1\right\}$:

$$\begin{array}{c} |(0,0)\rangle =|0\rangle \;|(0,1)\rangle =|+\rangle ,\\ |(1,0)\rangle =|1\rangle \;|(1,1)\rangle =|-\rangle . \end{array}$$

The protocol begins with the sender S preparing qubit states and sending them to the receiver R, which then samples a random string $\tilde{a}$. For every qubit received, R measures the *i*-th state on a computational basis if $\tilde{a_{i}}=0$ or, on the Hadamard basis, if $\tilde{a_{i}}=1$. Therefore, approximately half of R’s measurement results will be correlated with the prepared states by S, while the rest will be uncorrelated. To ensure security against a dishonest R, it is required to commit information on all of his measurement bases and outcomes to S, which then picks a random subset of them and tests for correlations. The passing of this test (statistically) ensures that R measured its qubits honestly. Next, S shares with R the bases it used for her state-preparation and, with this information, R knows which of its results are correlated with the sender’s. The receiver, then, creates two sets: $I_{0}$, with indices where it is measured on the same basis as S, and $I_{1}$, where their measuring bases differ. Following that, R uses its choice bit *b* to select the order in which it sends the two sets to S. The sender samples two hash functions $f_{0},f_{1}$ at random, from a *2-universal* family of hash functions F, in order to generate uniform keys of appropriate size, as that of the messages $m_{0},m_{1}$. S sends the encrypted messages $w_{0},w_{1}$ to R, which can only decrypt the message corresponding to the set $I_{0}$.

### 3.4. Post-Quantum UC-Secure ROT Protocol

**Figure 9:**
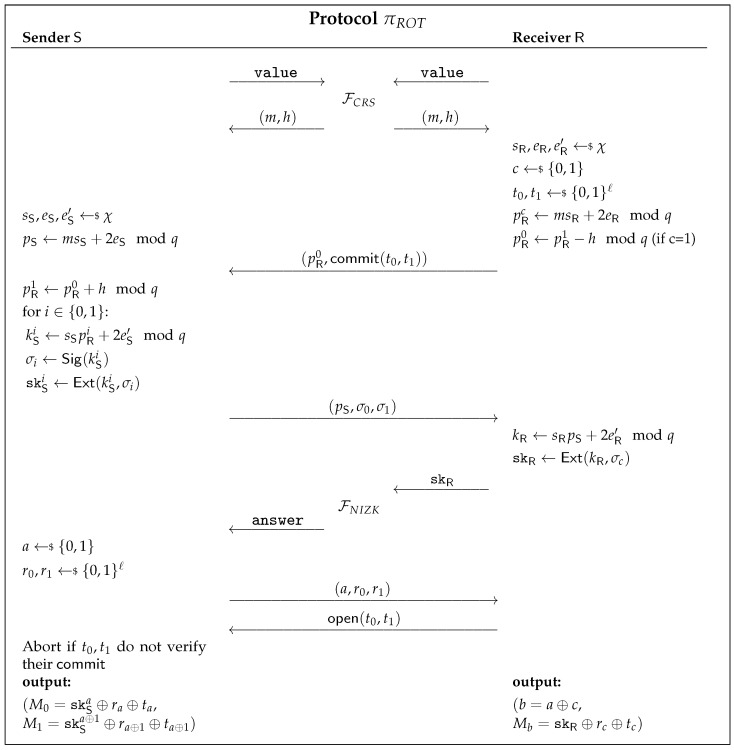
UC ROT protocol in the CRS model based on the RLWE assumption.

The protocol in Figure 9 is based on the recently proposed protocol by [30] (which was based on [29]), which has been shown to be UC-secure under the RLWE assumption in the Random Oracle Model (ROM). However, UC security using ROM does not directly lift to UC security against quantum adversaries. Taking that into consideration, our idea is to replace the random oracle calls, which are used to either commit to a string or to generate a random string.

In order to understand the protocol $\pi_{ROT}$, we need to provide some preliminary definitions. A signal function Sig and an extraction function Ext are described as in the key exchange protocol using RLWE of [34], to be used by the involved parties to reconcile a shared key.

Let $\sigma_{0},\sigma_{1}:Z_{q}\to \left\{0,1\right\}$. We define $\sigma_{0},\sigma_{1}$ as follows:

$$\sigma_{0}\left(a\right)=\left\{\begin{array}{cc} 0, & a\in [-\left\lfloor \frac{q}{4}\right\rfloor ,\lfloor \frac{q}{4}\rfloor ]\\ 1, & \mathrm{otherwise} \end{array}\right.\;\mathrm{and}\;\sigma_{1}\left(a\right)=\left\{\begin{array}{cc} 0, & a\in [-\lfloor \frac{q}{4}+1\rfloor ,\lfloor \frac{q}{4}+1\rfloor ]\\ 1, & \mathrm{otherwise} \end{array}\right.$$

Next, we need to extend $\sigma_{0},\sigma_{1}$ to the ring case. For any $a=\sum_{i=0}^{n-1}a_{i}X^{i}\in R_{q}$, we define $\sigma_{0},\sigma_{1}:R_{q}\to R_{2}$ as follows:

$$\sigma_{0}\left(a\right)=\sum_{i=0}^{n-1}\sigma_{0}\left(a_{i}\right)X^{i}\;\mathrm{and}\;\sigma_{1}\left(a\right)=\sum_{i=0}^{n-1}\sigma_{1}\left(a_{i}\right)X^{i}$$

The signal function $\mathrm{Sig}:R_{q}\to R_{2}$ can now be defined as $\mathrm{Sig}\left(a\right)=\sigma_{b}\left(a\right)$, where b←$ {0,1}, while the extraction function Ext: *R_q_* × *R*_2_ → *R*_2_ is

$$\mathrm{Ext}\left(a,\sigma \right)=\left(a+\sigma \frac{q-1}{2}\;\mathrm{mod}\;q\right)\;\mathrm{mod}\;2.$$

We can now describe the ROT protocol based on the RLWE assumption, Figure 9, which can be seen as a tweaked version of the protocol of [30], where we replace the random oracles by a commitment scheme and a NIZK protocol, modeled as functionalities.

Let *χ* and *q* be as in Definition 2 and *ℓ* be the security parameter. Let (m,h) be the common string, where $m,h\in R_{q}$, and let Ext and Sig be the algorithms defined above.

The protocol starts with both parties generating an RLWE sample. The sender S generates $p_{S}=ms_{S}+2e_{S}\;\mathrm{mod}\;q$, and the receiver R generates $p_{R}^{c}=ms_{R}+2e_{R}\;\mathrm{mod}\;q$, where *c* is a bit randomly chosen by R. If the sampled bit c=1, then R computes $p_{R}^{0}=p_{R}^{1}-h\;\mathrm{mod}\;q$. The receiver then samples two strings $t_{0},t_{1}\leftarrow \;\$\;{\left\{0,1\right\}}^{\ell }$ commits both strings, and sends $p_{R}^{0}$ to S. The sender uses the common string *h* and $p_{R}^{0}$ to compute $p_{R}^{1}=p_{R}^{0}+h$ mod *q* and uses both values $p_{R}^{0},p_{R}^{1}$ to generate two RLWE samples. $k_{S}^{i}=s_{S}p_{R}^{i}+2e_{S}^{\prime }$ mod *q* for i∈{0,1}. S now computes $\sigma_{i}=\mathrm{Sig}(k_{S}^{i})$ and ${\mathrm{sk}}_{S}^{i}=\mathrm{Ext}(k_{S}^{i},\sigma_{i})$, for i∈{0,1} and sends $p_{S},\sigma_{0},\sigma_{1}$ to R. The receiver then generates an RLWE sample $k_{R}=s_{R}p_{S}+2e_{R}^{\prime }$ mod *q* from $p_{S}$ and computes ${\mathrm{sk}}_{R}=\mathrm{Ext}(k_{R},\sigma_{c})$. The key exchange protocol guarantees that ${\mathrm{sk}}_{S}^{c}={\mathrm{sk}}_{R}$ with overwhelming probability, so as to guarantee that R did not cheat (and indeed the computed ${\mathrm{sk}}_{R}$). Both parties engage in a NIZK protocol. If the proof fails, S aborts; otherwise, he samples a bit *a* and two strings $r_{0},r_{1}\leftarrow \;\$\;{\left\{0,1\right\}}^{\ell }$ and sends *a*, *r*0, *r*1 to R. The receiver opens his initial commitment to S, and if the test passes, both parties output their messages: S outputs ($M_{0}={\mathrm{sk}}_{S}^{a}⊕r_{a}⊕t_{a},\;M_{1}={\mathrm{sk}}_{S}^{a⊕1}⊕r_{a⊕1}⊕t_{a⊕1}$), and R outputs ($b=a⊕c,\;M_{b}={\mathrm{sk}}_{R}⊕r_{c}⊕t_{c}$).

To simplify the description of $\pi_{ROT}$ in Figure 9, we represent $F_{NIZK}$ with a single input from the prover R (the witness *w*) and a single output to the verifier S, where this output is 1 if *w* satisfies R or 0 otherwise. Let the binary relation R be such that

$$R\left(x,w\right)=1\Leftrightarrow w={\mathrm{sk}}_{S}^{0}\vee w={\mathrm{sk}}_{S}^{1},$$

where $x=\mathrm{Enc}({\mathrm{sk}}_{S}^{0},{\mathrm{sk}}_{S}^{1})$ for a given public key encryption scheme.

The $F_{NIZK}$ functionality can, for instance, be instantiated using the protocol described in [35]. This protocol is shown to be quantum-composable in the CRS model, based on the LWE assumption.

## 4. Security

In this section, we establish the quantum-UC security of the proposed protocols in the CRS model. We begin by analyzing the quantum protocol first and proving that $\pi_{OT\to ROT}$ is quantum-UC-secure when instantiated with $\pi_{COM}$ and $\pi_{QOT}^{\pi_{COM}}$. We then prove the quantum-UC security of the $\pi_{ROT}$.

### 4.1. Quantum-UC Security of the Quantum ROT Protocol

**Theorem** **2.** *Protocol $\pi_{OT\to ROT}$ quantum-UC-emulates $F_{ROT}$ in the $\langle F_{OT},F_{COM}\rangle$-hybrid model.*

**Proof.**  We start by describing how the simulator S behaves in each of the possible cases for the execution of the protocol when an adversary A is present.

*Corrupted Sender.* In this case, S simulates the view of the sender, effectively controlling the inputs to $F_{COM}$ and the input bit to $F_{OT}$. In order to do so, we start by replacing $F_{COM}$ by a commitment functionality $F_{FakeCOM}$, which allows the receiver to cheat. In the commit phase, $F_{FakeCOM}$ expects a message commit instead of (commit, *x*); in the open phase, $F_{FakeCOM}$ expects a message (open, *x*) instead of open, which is then sent to the sender. We now change the receiver’s implementation to match with the new functionality; that is, when committing to message *m*, the receiver stores that message and later gives it to $F_{FakeCOM}$ when opening the commitment.

We can now describe how the simulator works. S starts by receiving $\left(M_{0},M_{1}\right)$ from $F_{ROT}$; afterwards, it sends commit to $F_{FakeCOM}$, samples c←$ {0,1}, and sends *c* to $F_{OT}$. Upon receiving *d*, the simulator extracts $w_{0},w_{1}$ from observing the sender’s call to $F_{OT}$ and computes $r_{d}=M_{0}⊕w_{d}$ and $r_{d⊕1}=M_{1}⊕w_{d⊕1}$. Finally, it sends (open, $\left(r_{1},r_{1}\right)$) to $F_{FakeCOM}$.

*Corrupted Receiver.* Now, S simulates the view of the receiver, controlling the input messages to $F_{OT}$. The simulator starts by receiving (b,M) from $F_{ROT}$. After receiving the commitment message, S extracts the strings $r_{0},r_{1}$ and the bit *c* from observing the receiver’s call to $F_{COM}$ and $F_{OT}$, respectively. It then computes $w_{c}=r_{c}⊕M$ and d=b⊕c and samples $w_{c⊕1}\leftarrow \;\$\;{\left\{0,1\right\}}^{\ell }$; afterwards, send $\left(w_{0},w_{1}\right)$ to $F_{OT}$ and *d* to A. When $F_{COM}$ replies with open $\left(r_{0},r_{1}\right)$, it checks if the values received match the original commitments and aborts if they do not.
*Both/None parties corrupted.* When both parties are corrupted, S internally runs A, which generates the messages for both parties.
   When the adversary does not corrupt any party, the simulator does not have an input from the ideal functionality $F_{ROT}$. As such, S runs the honest receiver and the honest sender, executing the needed algorithms when a dummy party is called in the ideal execution. The simulator forwards the messages of the honestly simulated protocol to A.

To finish the proof, it remains to show that the simulated executions of the protocol are indistinguishable from the real one.

**Claim** **1.** *If the adversary A corrupts the sender, then the real execution of the protocol $\pi_{OT\to ROT}$ is indistinguishable from the simulated one.*

**Proof.**  The real world execution can be viewed as a game that proceeds as follows:

1. Sample values $r_{0},r_{1}\leftarrow \;\$\;{\left\{0,1\right\}}^{\ell }$ and commit to values $r_{0},r_{1}$.
2. Sample bit c←$ {0,1} and run the OT protocol with the choice bit *c*.
3. Open the commitment to values $r_{0},r_{1}$.

The ideal world execution can be viewed as a game that proceeds as follows:

1. Send commit to $F_{FakeCOM}$.
2. Sample bit c←$ {0,1} send *c* to $F_{OT}$.
3. Send (open, $\left(r_{0},r_{1}\right)$) to $F_{FakeCOM}$, where $r_{d}=M_{0}⊕w_{d}$ and $r_{d⊕1}=M_{1}⊕w_{d⊕1}$.

The differences between the two traces are the commitment functionality and how the values $r_{0},r_{1}$ are generated. However, since the commitments are opened in the same way, replacing $F_{COM}$ by $F_{FakeCOM}$ leads to a perfectly indistinguishable network. Regarding $r_{0},r_{1}$, since $M_{0},M_{1}$ are uniform random values, which come from $F_{ROT}$, the values $r_{0},r_{1}$ are also statistically indistinguishable from uniform random values. Therefore, the two executions are statistically indistinguishable. □

**Claim** **2.** *If the adversary A corrupts the receiver, then the real execution of the protocol $\pi_{OT\to ROT}$ is indistinguishable from the simulated one.*

**Proof.**  The real world execution can be viewed as a game that proceeds as follows:

1. Sample strings $w_{0},w_{1}\leftarrow \;\$\;{\left\{0,1\right\}}^{\ell }$ and run the OT protocol with $w_{0},w_{1}$.
2. Sample bit *d* and send it to R
3. Check if the received values verify their commitment.

The ideal world execution can be viewed as a game that proceeds as follows:

1. Sample string $w_{c⊕1}\leftarrow \;\$\;{\left\{0,1\right\}}^{\ell }$ and compute $w_{c}=r_{c}⊕M$; afterwards, send ($w_{0},w_{1}$) to $F_{ROT}$.
2. Compute d=b⊕c and send it to R.
3. Check if the received values verify their commit.

In this case, the difference between both traces is in how $w_{c}$ and *d* are generated. Since *M* and *b* are uniform random values, which come from $F_{ROT}$, both the string $w_{c}$ and the bit *d* are statistically indistinguishable from a uniform random string and a uniform random bit, respectively. Thus, the above two executions are statistically indistinguishable. □

Finally, it is trivial to conclude that, when both parties are corrupted and when neither parties are corrupted, the simulated executions of the protocol are indistinguishable from the real execution. This concludes the proof. □

We have shown that, with $\pi_{OT\to ROT}$, we can transform $\pi_{QOT}$ into a ROT. We now need to prove that $\pi_{COM}$ remains UC-secure when working in a quantum setting.

**Theorem** **3.** *Let $G_{pk}$ be a quantum robust PRNG. $\pi_{COM}$ then (computationally) quantum UC-emulates $F_{COM}$ in the CRS model.*

**Proof.**  We start by briefly describing the UC security proof of $\pi_{COM}$ by Canneti in [33].

The simulation starts with the simulator S by generating $pk_{0},pk_{1}$, sampling random $r_{0},r_{1}\in {\left\{0,1\right\}}^{n}$, and setting $\sigma =G_{pk_{0}}\left(r_{0}\right)⊕G_{pk_{1}}\left(r_{1}\right)$. With this fake string, S tells the adversary A that the sender is committed to $y=G_{pk_{0}}\left(r_{0}\right)$. By later sending $r_{0}$ or $r_{1}$, the simulator is able to open the commitment to either b=0 or to b=1, respectively. If it were possible to distinguish the fake string from the real one, it would contradict the pseudo-randomness of the generator.

When working in a quantum setting, the indistinguishability of the fake string reduces to the pseudo-randomness of the generator; that is, the environment can only distinguish between the real world and ideal world executions if it is possible to distinguish the fake string *σ* from the real one. As such, if the generators are quantum robust, the environment will not be able to distinguish between both strings. Therefore, the arguments used in the classical UC security proof follow for quantum UC security as well. □

Finally, we analyze the security of the proposed composition of protocols. Let $\pi_{QROT}$ denote $\pi_{OT\to ROT}$ instantiated with $\pi_{COM}$ and $\pi_{QOT}^{\pi_{COM}}$.

**Theorem** **4.** *Protocol $\pi_{QROT}$ quantum-UC-emulates $F_{ROT}$.*

**Proof.**  First, we analyze the UC security of $\pi_{QOT}^{\pi_{COM}}$. Protocol $\pi_{QOT}$ with ideal commitments is known to be universally composable [28]; as such, since $\pi_{COM}$ is a composable commitment scheme, we have that $\pi_{QOT}^{\pi_{COM}}$ quantum-UC-emulates $F_{OT}$.

Finally, as was shown in Theorem 2, $\pi_{OT\to ROT}$ with ideal commitments and an ideal OT is universally composable. Since both $\pi_{COM}$ and $\pi_{QOT}^{\pi_{COM}}$ are universally composable, the result follows directly. □

A downside of using $\pi_{COM}$ as the commitment scheme is that we require a call to $\pi_{COM}$ for each bit of the string we intend to commit, which will affect the protocol’s efficiency. However, since a composable commitment is required, this is our best suggestion in the CRS model.

### 4.2. Quantum-UC Security of the Post-Quantum ROT Protocol

We now analyze the security of $\pi_{ROT}$. The simulator will use its ability to program the CRS and extract the NIZK witness in order to obtain the desired UC security.

**Theorem** **5.** *Protocol $\pi_{ROT}$ (computationally) quantum-UC-emulates $F_{ROT}$ in the CRS model, given that the HNF-RLWE assumption holds.*

**Proof.**  Once again, we describe the behavior of the simulator S in each of the possible cases for the execution of the protocol when an adversary A is present.

*Corrupted Sender.* The simulator S simulates the view of the sender, meaning that it controls the communication with R as well as the inputs of $F_{COM}$ and $F_{NIZK}$. As in the proof of security for $\pi_{QROT}$, we will be replacing $F_{COM}$ by the functionality $F_{FakeCOM}$ and changing the receiver’s implementation to match $F_{FakeCOM}$.

S starts by receiving $\left(M_{0},M_{1}\right)$ from $F_{ROT}$. It then samples c←$ {0,1} and $t_{0},t_{1}\leftarrow \;\$\;{\left\{0,1\right\}}^{\ell }$, as an honest receiver would. Next, it computes two RLWE samples, $p_{R}^{0}=ms_{R}^{0}+2e_{R}^{0}$ mod *q* and $p_{R}^{1}=ms_{R}^{0}+2e_{R}^{0}$ mod *q*, sets $h=p_{R}^{1}-p_{R}^{0}$, and programs $F_{CRS}$ to return (m,h) when queried. Following that, it sends $p_{R}^{0}$ to A and sends commit to $F_{FakeCOM}$.

After receiving $\left(p_{S},\sigma_{0},\sigma_{1}\right)$, S computes ${\mathrm{sk}}_{R}^{i}=\mathrm{Ext}\left(s_{R}^{i}p_{S}+2{e_{R}^{\prime }}^{i},\sigma_{i}\right)$, for i∈{0,1}, and sends ${\mathrm{sk}}_{R}^{c}$ to $F_{NIZK}$. Finally, upon receiving $a,r_{0},r_{1}$, S computes $t_{a}=M_{0}⊕{\mathrm{sk}}_{S}^{a}⊕r_{a}$ and $t_{a⊕1}=M_{1}⊕{\mathrm{sk}}_{S}^{a⊕1}⊕r_{a⊕1}$ and sends (open, $\left(t_{0},t_{1}\right)$) to $F_{FakeCOM}$.

*Corrupted Receiver.* In this case, S simulates the view of the receiver, controlling the communication with S. The simulator starts by receiving (b,M) from $F_{ROT}$. It computes $p_{S}$ as an honest sender; after receiving $p_{R}^{0}$ as well as the receipt of the commitment, it computes ${\mathrm{sk}}_{S}^{i},\sigma_{i}$ honestly, for i∈{0,1}, and sends $p_{S},\sigma_{0},\sigma_{1}$ to A. After receiving the reply from $F_{NIZK}$, if the test passed, S extracts *c* from observing the call made to $F_{NIZK}$ and comparing ${\mathrm{sk}}_{R}$ to ${\mathrm{sk}}_{S}^{0}$ and ${\mathrm{sk}}_{S}^{1}$. Finally, it computes a=b⊕c and $r_{c}=M⊕{\mathrm{sk}}_{S}^{c}⊕t_{c}$, samples $r_{c⊕1}\leftarrow \;\$\;\left\{0,1\right\}$ and sends $a,r_{0},r_{1}$ to A. At the end, it checks if $t_{0},t_{1}$ match the initial commitment, aborting if they do not.
*Both/None parties corrupted.* Here, both cases work as in the previous UC security proof. When both parties are corrupted, the adversary is ran internally by S. When neither of the parties are corrupted, S runs the honest receiver and sender, sending all the messages between them to A.

Again, we now need to show that the real execution of the protocol is indistinguishable from the simulated ones.

**Claim** **3.** *If the adversary A corrupts the sender, then the real execution of the protocol $\pi_{ROT}$ is indistinguishable from the simulated one.*

**Proof.**  The real world execution can be viewed as a game that proceeds as follows:

1. Sample bit c←$ {0,1} and strings $t_{0},t_{1}\leftarrow \;\$\;{\left\{0,1\right\}}^{\ell }$.
  Generate RLWE sample $p_{R}$ and, if c=1, compute $p_{R}^{0}=p_{R}^{1}-h$.
  Send $p_{R}^{0}$ and commit to values $t_{0},t_{1}$.
2. Compute ${\mathrm{sk}}_{R}=\mathrm{Ext}\left(s_{R}p_{S}+2e_{R}^{\prime },\sigma_{c}\right)$ and run the NIZK protocol with ${\mathrm{sk}}_{R}$.
3. Open the commitment to values $t_{0},t_{1}$.

The ideal world execution can be viewed as a game that proceeds as follows:

1. Sample bit c←$ {0,1}.
  Generate RLWE samples $p_{R}^{0},p_{R}^{1}$ and program $F_{CRS}$ to return $\left(m,p_{R}^{1}-p_{R}^{0}\right)$.
  Send $p_{R}^{0}$ to A and send commit to $F_{FakeCOM}$.
2. Compute ${\mathrm{sk}}_{R}^{i}=\mathrm{Ext}\left(s_{R}^{i}p_{S}+2{e_{R}^{\prime }}^{i},\sigma_{i}\right)$, for i∈{0,1}, and send ${\mathrm{sk}}_{R}^{c}$ to $F_{NIZK}$.
3. Send (open,$\left(t_{0},t_{1}\right)$) to $F_{FakeCOM}$, where $t_{a}=M_{0}⊕{\mathrm{sk}}_{S}^{a}⊕r_{a}$ and $t_{a⊕1}=M_{1}⊕{\mathrm{sk}}_{S}^{a⊕1}⊕r_{a⊕1}$.

The first difference between both games is in $p_{R}^{0}$ and $p_{R}^{1}$. In the real world game, only $p_{R}^{c}$ is an RLWE sample ($p_{R}^{c⊕1}$ is a uniform random sample), while in the ideal world game, both $p_{R}^{0}$ and $p_{R}^{1}$ are RLWE samples. Given that the RLWE assumption holds, both situations are indistinguishable.

Once again, replacing $F_{COM}$ by $F_{FakeCOM}$ leads to an indistinguishable network, since the commitments are opened in the same way. Finally, in the real world, $t_{0},t_{1}$ are uniform random values, while in the ideal world, they are not. However, since $M_{0},M_{1}$ are uniform random values that come from $F_{ROT}$, the values in the ideal world are statistically indistinguishable from uniform random values.

Thus, the two executions are indistinguishable, assuming the RLWE assumption holds. □

**Claim** **4.** *If the adversary A corrupts the receiver, then the real execution of the protocol $\pi_{ROT}$ is indistinguishable from the simulated one.*

**Proof.**  The real world execution can be viewed as a game that proceeds as follows:

1. Generate RLWE sample $p_{S}$.
2. Compute $p_{R}^{1}=p_{R}^{0}+h\;\mathrm{mod}\;q$. Compute $\sigma_{i}$ and ${\mathrm{sk}}_{S}^{i}$, for i∈{0,1}.
  Send $\left(p_{S},\sigma_{0},\sigma_{1}\right)$.
3. Run the NIZK protocol and check if the test passes; abort if it does not.
  Sample a←$ {0,1} and $r_{0},r_{1}\leftarrow \;\$\;{\left\{0,1\right\}}^{\ell }$. Send ($ar_{0},r_{1}$).
4. Check if the received values verify their commitment; abort if they do not.

The ideal world execution can be viewed as a game that proceeds as follows:

1. Generate RLWE sample $p_{S}$.
2. Compute $p_{R}^{1}=p_{R}^{0}+h\;\mathrm{mod}\;q$. Compute $\sigma_{i}$ and ${\mathrm{sk}}_{S}^{i}$, for i∈{0,1}.
  Send $\left(p_{S},\sigma_{0},\sigma_{1}\right)$.
3. Check if the received answer from $F_{NIZK}$ is 1; abort if it is not.
  Send $\left(a,r_{0},r_{1}\right)$, where a=b⊕c, $r_{c}=M⊕{\mathrm{sk}}_{S}^{c}⊕t_{c}$, and $r_{1-c}\leftarrow \;\$\;{\left\{0,1\right\}}^{\ell }$.
4. Check if the received values verify their commitment; abort if they do not.

The games differ in how *a* and $r_{c}$ are generated; however, since *b* and *M* are uniform random values that come from $F_{ROT}$, both $r_{c}$ and *a* are statistically indistinguishable from a uniform random string and a uniform random bit, respectively. Hence, the real world execution and the ideal world execution are indistinguishable, assuming that the RLWE assumption holds. □

It remains to be seen whether the simulated executions where both parties are corrupted and when no party is corrupted are also indistinguishable. As in the previous proof, both are trivial, which concludes the proof. □

## 5. Conclusions

In view of the usefulness of MPC and the steady evolution of both quantum technology and post-quantum cryptography techniques, as well as recognizing the potential threat quantum computers can present in the landscape of information security, we have proposed two potential solutions for quantum secure implementations of ROT.

Both of these protocols have in common that they use a commitment scheme based on quantum-secure pseudo-random generators, which is universally composable in the CRS model. The CRS assumption has the advantage of being weaker and better understood than the quantum random oracle, and it is independent of technological limitations as opposed to the noisy storage assumptions, which are two of the most common models in which the security of OT protocols is studied.

The first construction is based on a quantum OT protocol composed with a quantum secure bit commitment, which is then transformed into a ROT protocol. The usage of a PRNG, which is secure against any poly-time quantum distinguisher, is the key to the commitment scheme’s quantum composability. The second construction is based on a highly efficient UC-secure ROT protocol from the RLWE assumption, initially proposed in the ROM. Our protocol differs in that we remove the random oracle’s requirement, replacing it by a commitment scheme and non-interactive zero knowledge protocol, which allows us to make a quantum-secure UC protocol, but in the CRS model instead.

Potential future work directions include the following:

- Further optimization of the commitment scheme to reduce the number of CRS calls and PRNG computations per committed bit in the context of a string commitment scheme.
- The implementation of both protocols and a comparison of their performance, taking available (quantum) technologies into account. This poses a challenge, as the limitations of quantum technologies are much less known than traditional computational power and communication.

## Data Availability

Not applicable.
